# Prognostic Value of Tumor Deposits in Patients with Colorectal Cancer

**DOI:** 10.7150/jca.96655

**Published:** 2024-07-09

**Authors:** Manuel Díez Alonso, Fernando Mendoza Moreno, Cristina Vera Mansilla, Belén Matías, Rubén Jimenez, Youssef Allaoua, Ignacio Busteros Moraza, Alberto Vilar, Silvestra Barrena, Raúl Díaz-Pedrero, Miguel A Ortega, Melchor Alvarez de Mon, Alberto Gutierrez

**Affiliations:** 1 Department of General and Digestive Surgery, University Hospital Príncipe de Asturias, 28805 Madrid, Spain.; 2 Department of Surgery, Medical and Social Sciences. Faculty of Medicine and Health Sciences, University of Alcalá, 28801 Alcalá de Henares, Spain.; 3 Ramón y Cajal Institute of Sanitary Research (IRYCIS), University Hospital Príncipe de Asturias, 28034 Madrid, Spain.; 4 Department of Medicine and Medical Specialities, (CIBEREHD), Faculty of Medicine and Health Sciences, University of Alcalá, 28801 Alcalá de Henares, Spain.; 5 Department of Nursing and Physiotherapy, Faculty of Medicine and Health Sciences, University of Alcalá, 28801 Alcalá de Henares, Spain.; 6 Immune System Diseases-Rheumatology and Internal Medicine Service, University Hospital Príncipe de Asturias, (CIBEREHD), 28806 Alcalá de Henares, Spain.

**Keywords:** tumor nodules, colorectal cancer, cancer-related survival, time to recurrence, prognostic factors

## Abstract

Tumor nodules or tumor deposits (TDs) are a histopathological prognostic factor that are associated with a negative evolutionary course in patients with colorectal cancer (CRC). There are still controversial aspects of TDs, including how they should be integrated into the TNM classification system. The objective of this study was to analyze the predictive value of TDs for cancer-related survival (CRS) and time-to-recurrence survival (TTR) and to evaluate the prognostic value of TDs in patients whose tumors also presented lymph node metastasis (LNM). In this retrospective observational study, all patients treated for CRC between January 2010 and December 2020 at the same hospital were included. CRS and TTR were classified by tumor stage. The results were compared between patients whose tumors had TDs and patients whose tumors did not. A total of 1426 patients met the criteria for inclusion in the analysis. TDs were detected in 178 patients (12.5%): 60 had tumors without LNM, and 118 had LNM. Patients with TD tumors had a lower CRS at 60 months after diagnosis (42% vs. 82%; p < 0.001) and a shorter TTR (34% vs. 79%; p < 0.001). Cox multiple regression analysis revealed that the presence of TD was associated with an increased risk of death from CRC (HR: 1.820; 95% CI: 1.327-2.496) and an increased risk of recurrence (HR: 2.315; 95% CI: 1.743-3.073). In each N stage category, the CRS was significantly lower in the subgroup with TD^+^: in patients with N1a tumors, the CRS was 44% when TD^+^ vs. 70% when TD^-^ (p = 0.019); in the N1b group it was 36% vs. 66% (p < 0.001); in the N2a group it was 34% vs. 58% (p = 0.012); and in N2b tumors it was 23% vs. 53% (p = 0.031). The present study shows that the information on the presence of TDs is complementary to that provided by LNM and allows the identification of subgroups of patients in each N stage determined by two metrics, CRS and TTR. TDs should be included in the definition of TNM system categories in patients who simultaneously present with LNM.

## Introduction

Colorectal cancer (CRC) has a high incidence and mortality rate, being one of the leading causes of death from cancer worldwide. There is particular interest in the identification of clinical or histopathological factors that could reflect its evolutionary course and help us estimate the prognosis of survival or recurrence.

The tumor-node-metastasis (TNM) classification system developed by the American Joint Committee on Cancer (AJCC) and the Union for International Cancer Control is the most widely used system for describing the extent of disease, classifying patients and guiding treatment. The 5th edition of the AJCC TNM staging system, published in 1997, recognized the prognostic value of certain histopathological characteristics of tumors among which the tumor nodules or tumor deposits (TDs) was adopted as a staging factor 1. The definition of TD has been progressively modified as these have been studied in a more systematic way. Initially, they were considered extensions of the primary tumor in the pericolic fat, regardless of their histology, and were included in category T 2. In the 7th edition of the AJCC TNM staging system, published in 2009, a histological criterion was used for its definition: "irregular contour and no evidence of residual lymphatic tissue", and was included in category N. In addition, a new subcategory was introduced, named N1c 3. Patients with stage I or II tumors, with TDs and without regional lymph node metastasis (LNM), were included in this subcategory. This update signifies the recognition of the predictive value for the evolutionary course that TDs bring and was made to indicate the need to administer postoperative adjuvant chemotherapy in this group of patients 2,4. The AJCC 8th edition, published in 2017, defined TDs more restrictively, as “nodules without histological evidence of residual lymph node or identifiable vascular or neural structure”, though the pN1c subcategory remained unchanged 4.

Various publications have analyzed the association between the presence of TDs and the evolutionary course of the disease. Patients in whom TDs are detected have a lower overall survival (OS) and a lower disease-free survival (DFS) 5-9. The controversial aspects of TDs include their poorly understood biological significance, the predictive weight associated with their presence, and the effect of the coexistence of LNM. Therefore, the way in which these systems should be integrated into the TNM classification system has been discussed. In the current TNM system, TDs are not considered if they coexist at the same time as LNM. Patients with tumors classified as N1a, N1b, or N2 did not have their classification modified if a TD was detected. However, several publications have reported that patients with stage N1 or N2 tumors exhibit worse disease progression if they present with both LNM and TDs 10-13. Clinicians doubt how they should interpret the prognostic role of TDs in patients with CRC.

The objective of this study was to analyze the prognostic value of TDs for cancer-related survival (CRS) and time-to-recurrence survival (TTR) and to evaluate the prognostic value of TDs in patients whose tumors also present LNM.

## Material and Methods

This was a retrospective observational study. All patients treated for colorectal adenocarcinoma between January 2010 and December 2020 in the General Surgery Department of Hospital Universitario Príncipe de Asturias, Alcala de Henares, Madrid, Spain, were included. The main objective of this study was to analyze the prognostic value of TDs for CRS and TTR in patients with CRC. Survival was calculated for all patients who met the inclusion criteria, and the results were compared between patients with and without TDs. The study adhered to the STROBE guidelines for designing and reporting observational studies. Patients were identified from the computerized database of the Coloproctology Unit, which was prospectively developed during these years. The study was approved by the Ethics Committee of Hospital Príncipe de Asturias (Code: OE 37/2021).

### Patients and data

**Figure 1:**
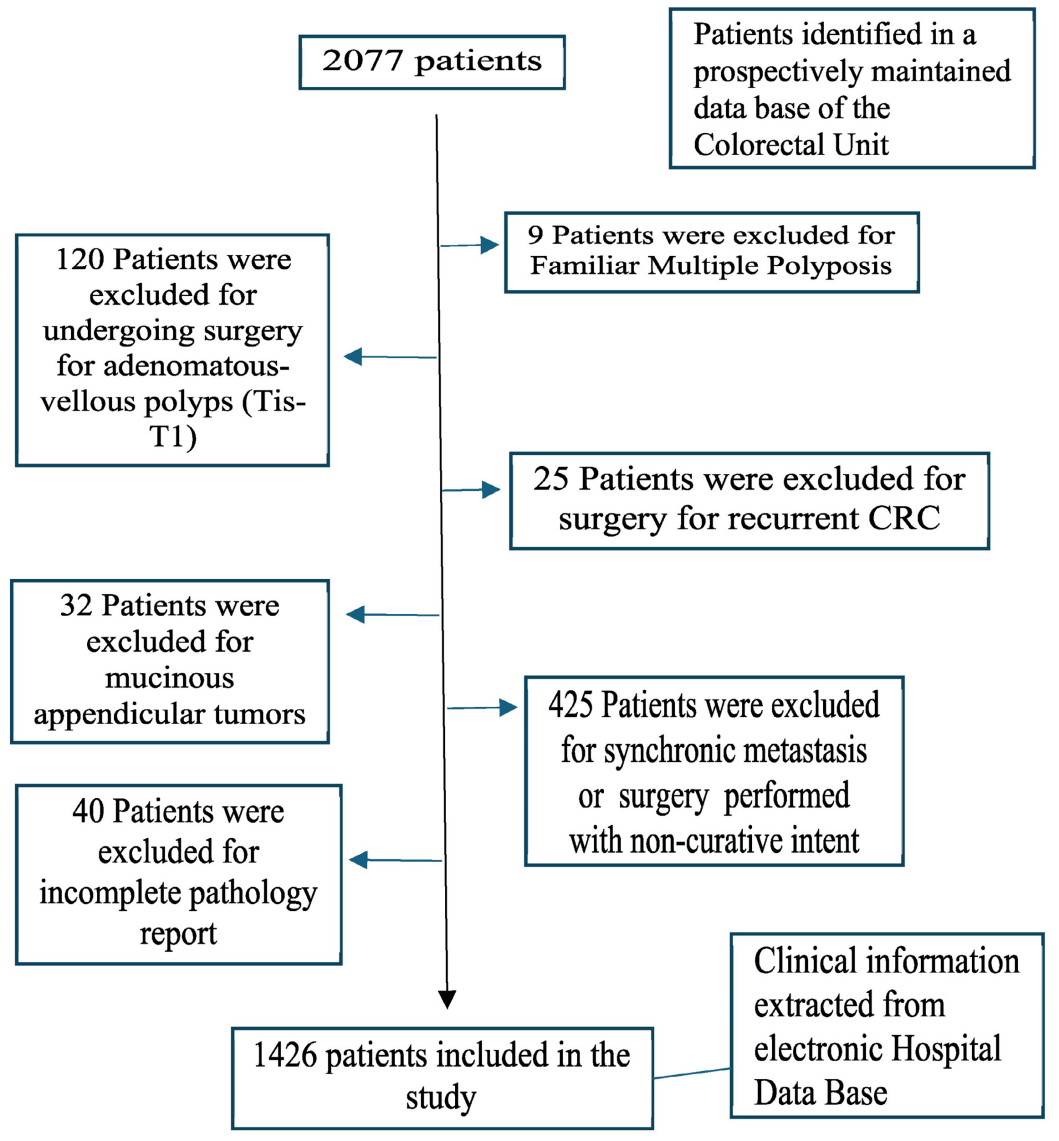
Flowchart detailing the selection of the patients in this study.

The inclusion criteria were as follows: age over 18 years, histopathology of the primary tumor compatible with colorectal adenocarcinoma, and curative surgical resection of the primary tumor. The exclusion criteria were familial multiple polyposis, adenomatous polyps or tumors in situ, recurrent CRC, mucinous appendicular tumors, surgery performed with noncurative intent, synchronous metastasis, and incomplete pathology (Figure 1).

After the diagnosis of CRC, all patients were evaluated by a multidisciplinary medical committee that assessed the possible therapeutic options according to the grade of extension, presence of metastasis in other organs, presence of local complications produced by the tumor, and functional status of the patient.

The clinical data of the patients were obtained from the electronic medical records of the hospital and were stored in an electronic database. The following data related to coincident predictor variables were collected: demographic information (sex and age), location of the primary tumor, surgical procedures, postoperative complications, medical oncological treatment received, and long-term outcome. CRC was staged according to the 8th edition of the TNM classification of the AJCC 4. Patients with tumors located in the cecum, ascending colon, hepatic angle, or transverse colon were classified as right-sided tumors; those with tumors originating in the splenic flexure, descending colon, or sigmoid colon were classified as left-sided tumors; and those located in the proximal 15 cm of the anus were classified as rectal tumors. There were no missing data for any of the variables that were included in the analysis.

Pathology was reviewed by a gastrointestinal pathologist in order to evaluate the presence of TDs, tumor grade, degree of differentiation, histological type, mucinous component, number of lymph nodes examined, number of lymph nodes metastasized, degree of local tumor infiltration, and extent of perineural or lymphovascular infiltration. TDs were defined as “macroscopic or microscopic nest of cancer, in the pericolorectal adipose tissue`s lymph drainage area of a primary carcinoma, discontinuous from the primary and without histological evidence of residual lymph node or identifiable vascular or neural structure”, as indicated by the 8th edition of the TNM classification of the AJCC 4,5.

After the initial treatment, the patients were followed up in accordance with the current guidelines by means of physical examination, analytical assessment every 6 months during the first 2 years and annually thereafter, annual computed tomography (CT) scans up to the 5th year, and colonoscopy 1 and 3 years after surgery.

### Main outcome measures

The primary outcomes of interest were CRS and TTR. Survival was estimated in months from the date of diagnosis to the date of death or the last date of follow-up. To calculate CRS, deaths due to colorectal cancer were considered deaths, and those due to another cause were censored. The TTR was defined as the time from diagnosis to the time of recurrence. Patients with no disease recurrence were censored at the last time when they were known to be recurrence free.

### Statistical analysis

The variables were input into a Microsoft Excel 2019 (v.27) (Microsoft, Redmond, WA, USA) spreadsheet. Statistical analysis was performed with SPSS (v.23) (IBM, Armonk, New York, NY, USA).

Initially, the distributions of clinical and histopathological characteristics among patients with tumors with TDs (TD^++^) and tumors without TD (TD^-^) were compared using the χ-squared test. Next, survival up to 60 months after diagnosis and median survival for each variable included in the present study were analyzed using the Kaplan‒Meier estimator. The log-rank test was used to compare survival curves.

Finally, the effect of each variable on survival was evaluated using Cox proportional-hazard regression. Cox regression models were built using the backward method. Variables included in the adjusted models were those that had p < 0.05 for the outcome of interest in the univariate analysis. These variables were kept in the final model if they were still significant at p < 0.05. The assumption of proportional hazards across different covariates was tested by inspecting the log (-log) plots. The risk of death or recurrence was expressed as the hazard ratio (HR) with its 95% confidence interval (CI). To avoid collinearity in this analysis, the two factors that make up the N stage, LNM and TD, were studied separately.

## Results

### Patients and characteristics

A total of 1426 patients met the criteria for inclusion. There were 884 (62%) men and 542 (38%) women. The mean age was 68 ± 11 years (range: 69). The mean follow-up was 56 ± 34 months (median: 51). The tumor was located in the right colon in 471 (33%) patients, in the left colon in 576 (40.4%) patients and in the rectum in 379 (26.6%) patients. Overall, 353 (24.8%) patients had TNM stage I tumors, 582 (40.8%) had stage II tumors, and 491 (34.4%) had stage III tumors. Table 1 shows the distribution of the clinical and histopathological characteristics of the patients.

### Patient and tumor characteristics categorized by the detection of tumor depth

**Figure 2:**
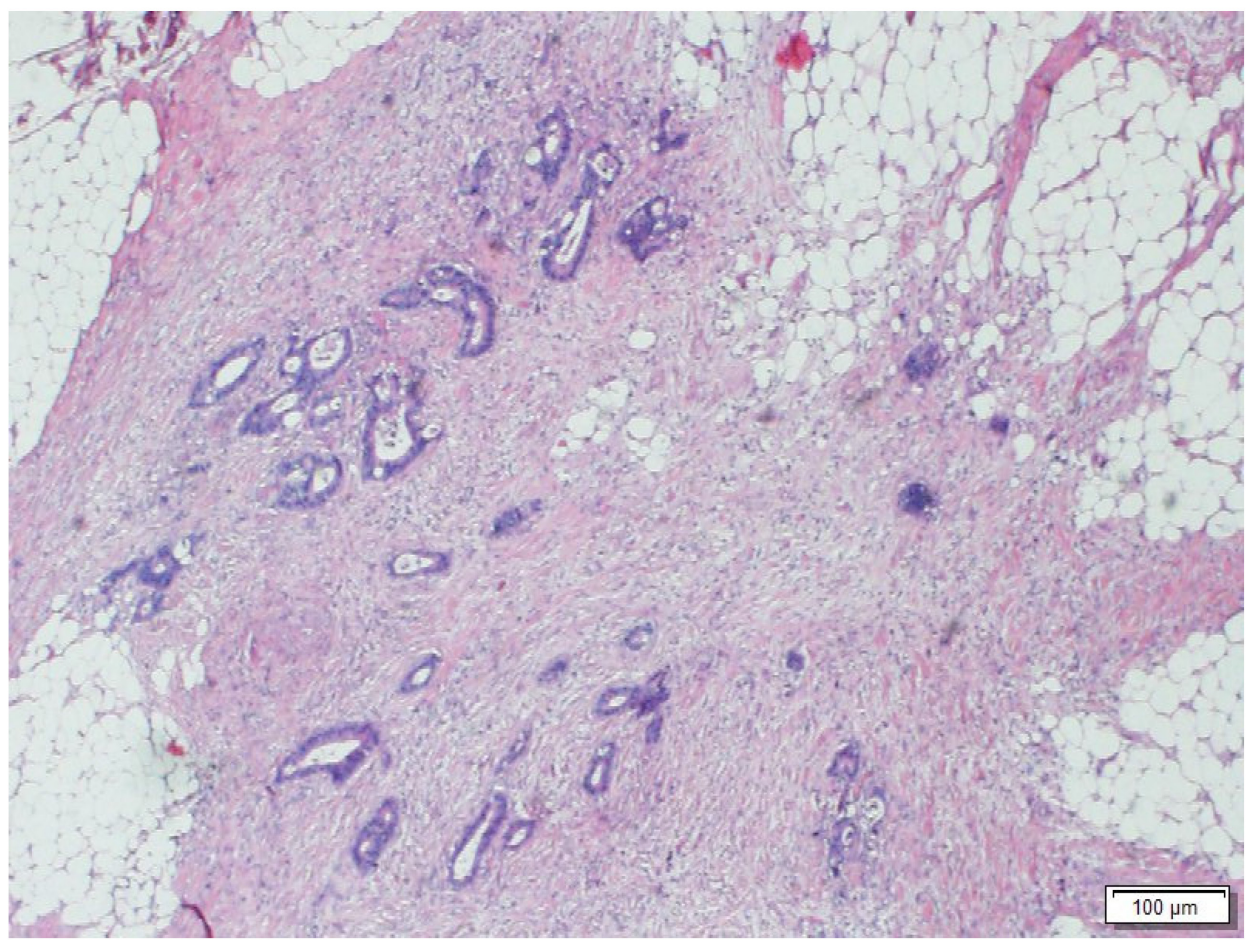
Morphologic image of a Tumor Deposit. Neoplastic cells infiltrating pericolic adipose tissue. Hematoxylin and Eosin staining, 40 X.

TDs were detected in 178 patients (12.5%) (Figure 2). Of these, 60 (33.7%) had tumors that did not present LNM, while in 118 (66.3%) the TD coexisted with LNM. The incidence of TD increased as the number of LNMs increased (6.1% in LNM-negative tumors, 18.8% in tumors with 1-3 LNMs and 51.3% in tumors with > 3 LNMs; p < 0.001).

The incidence of TDs did not differ by sex, age, location of the primary tumor, or histologic type. TDs were associated with higher T stage (0.6% in T1, 2.8% in T2, 13.7% in T3 and 31.1% in T4; p < 0.001), poorly differentiated tumors (26.6 vs. 11.3%; p < 0.001), lymphovascular infiltration (39.3 vs. 7.5%; p < 0.001), perineural infiltration (38.3 vs. 8.3%; p < 0.001), tumors that presented intestinal obstruction (18 vs. 12%; p = 0.041), and perforated tumors (30.7 vs. 12.6%; p < 0.0.001). Among the 178 tumors with TDs, 88 (49.3%) also had lymphovascular infiltration, and 77 (43.2%) also had perineural infiltration.

### Cancer-related survival

During follow-up, 262 patients died due to CRC. Kaplan-Meier estimates of CRS at 60 months after diagnosis for the entire cohort were 77%. CRS at 60 months was lower in patients with TD (42% vs. 82%; p < 0.001) (HR: 4.497; 95% CI: 3.466-5.835). The results of the univariate survival analysis are shown in Table 2, including all the clinical and histopathological variables analyzed.

Other factors that were significantly associated with survival were T stage (96% in T1, 89% in T2, 76% in T3, 48% in T4; p <p.001), Lymph Node Metastasis (87% in tumors with 0 LNM, 63% in tumors with 1-3 LNM and 39% in tumors with more than 3 LNM; p< 0.001), presence of intestinal obstruction (62 vs. 78%; p< 0.001), presence of tumor perforation (46 vs. 79%; p< 0.001), lymphovascular infiltration (51 vs. 82%; p< 0.001), perineural infiltration (56 vs. 81%; p< 0.001), poor grade of differentiation (60 vs. 78%; p.001), and mucinous histologic type (71 vs. 78%; p=0.015).

### CRS categorized by the presence of tumor deposits and lymph node metastases

**Figure 3:**
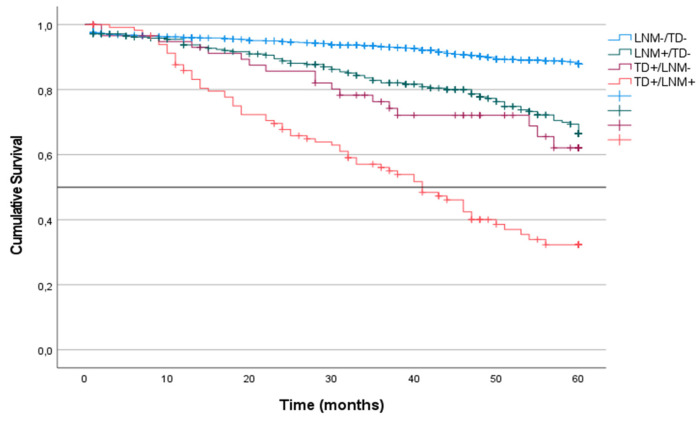
Kaplan-Meier estimates of CRS for the entire cohort according to the presence of Tumor Deposit and Lymph Node Metastasis. Horizontal bar denotes median survival.

CRS was analyzed in the four groups of patients in the cohort. CRS incidence was defined according to the presence or absence of LNM or TD without considering other coinciding factors. Patients with TD^-^ or LNM^-^ tumors had the highest survival at 60 months of follow-up (87%). CRS was similar in patients with LNM^+^/TD^-^ and in patients with TD^++^/LNM^-^ (62% and 63%, respectively). In contrast, patients with TD^++^/LNM^+^ tumors had the lowest survival (36%; median: 41) (p < 0.001) (Figure 3).

Next, CRS was analyzed in the three subgroups of patients included in the N1 subgroup according to the 8th edition of the AJCC TNM system. Survival was higher in the N1a subgroup (67%) than the N1b (57%) and N1c subgroups (63%), although the difference between them was not statistically significant (p = 0.086).

**Figure 4:**
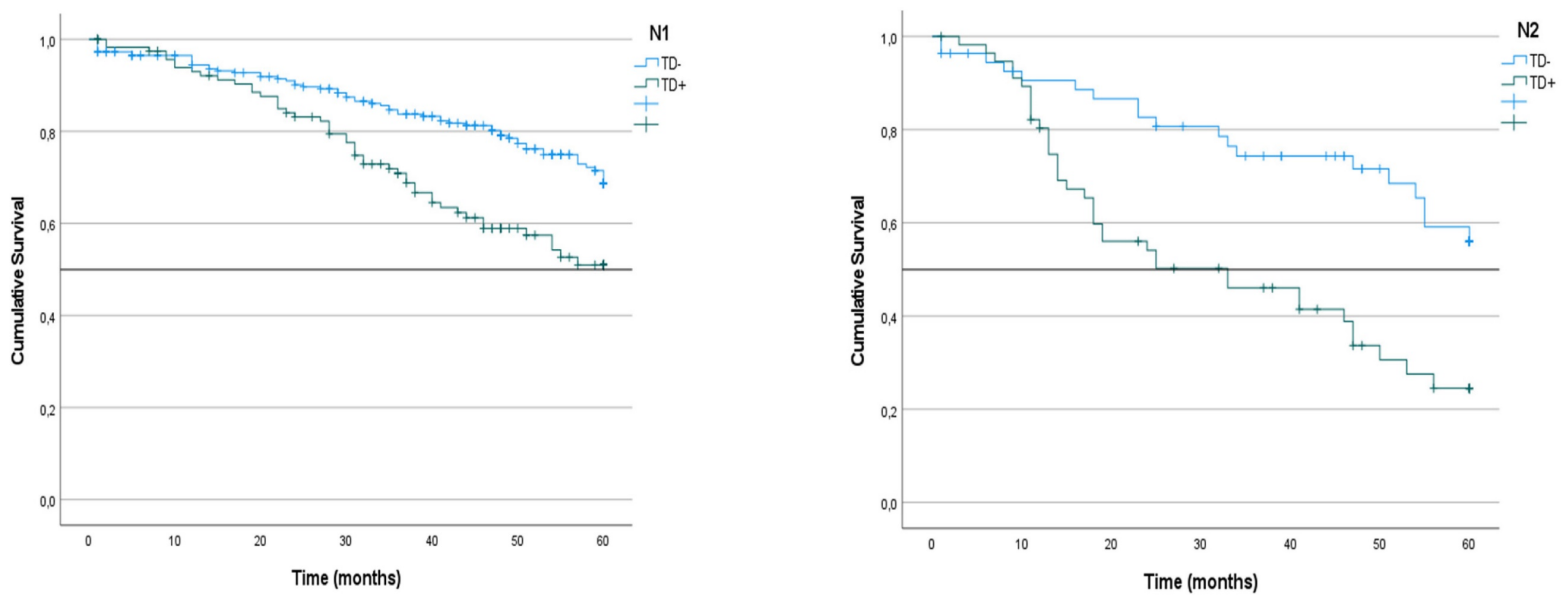
Kaplan-Meier estimates of CRS in N1 and N2 tumors according to presence of Tumor Deposit. Horizontal bars denote median survival.

**Figure 5:**
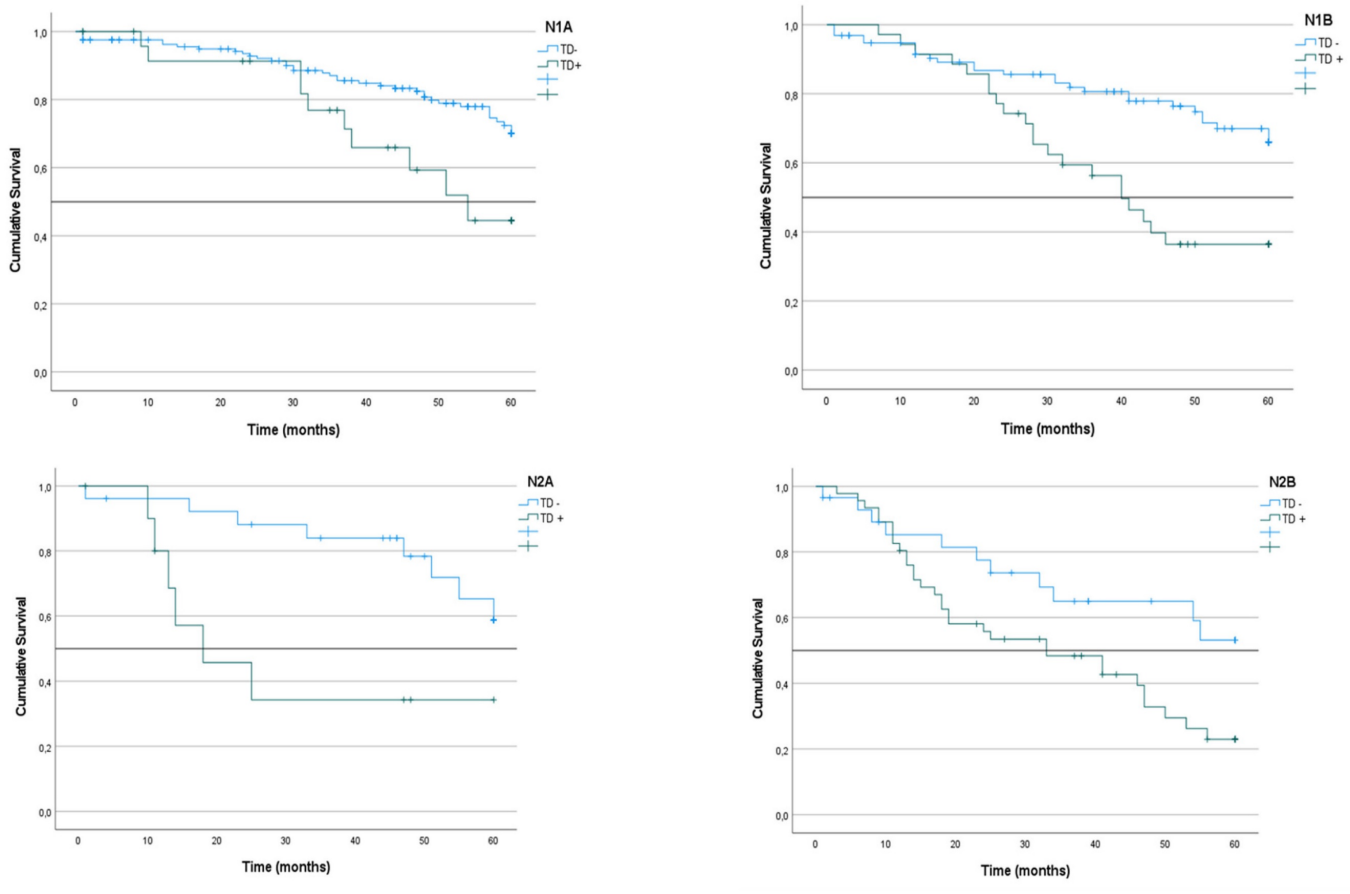
Kaplan-Meier estimates of CRS in N1a, N1b, N2a and N2b tumors according to presence of Tumor Deposit. Horizontal bars denote median survival.

We analyzed the CRS of patients with LNM classified according to the presence or absence of TDs. In the group of patients with N1 tumors, survival was lower among those with TD^+^ tumors than among those with TD^-^ tumors (51% vs. 68%; p < 0.001) (Figure 4). The same difference was seen in the subgroups with TD^+^ N1a tumors (44% vs. 70%; p = 0.019) and TD^+^ N1b tumors (36% vs. 66%; p < 0.001) (Table 3) (Figure 5). Similarly, in the group of patients with N2 tumors, CRS was lower among the patients with TD^++^ tumors (25% vs. 56%; 0 < 0.001) (Figure 3), including in the subgroups of patients with N2a tumors TD^+^ (34% vs. 58%; p = 0.012) and N2b tumors (23% vs. 53%; p = 0.031) (Table 3) (Figure 4).

According to our multiple regression analysis, the presence of TDs had a significant adverse effect on CRS (HR: 1.820; 95% CI: 1.397-2.496; p< 0.001) (Table 4). The other factors that showed an independent predictive value are listed in Table 4. The negative effect of LNM on the prognosis of survival had a HR of 2.661 (95% CI: 1.804-3.926) in patients with 1-3 vs. 0 LNMs and 1.961 (95% CI: 1.466-2.622) in patients with > 3 vs. 1-3 LNMs.

In addition, we analyzed separately the effect of TDs on CRS in the group of TNM Stage III patients (Table 5). The multiple regression analysis, showed that in this group TDs had, also, a significant adverse effect on CRS (HR: 2.040; 95% CI: 1.411-2.950).

### Time to recurrence

During follow-up, 320 (22.4%) patients experienced tumor recurrence. The Kaplan-Meier estimate of TTR at 60 months after diagnosis for the entire cohort was 73%. TTR at 60 months was lower in patients with TD^+^ tumors than in patients with TD^-^ tumors (34% vs. 79%; p < 0.001) (HR: 5.172; 95% CI: 4.082-6.551). The results of the univariate survival analysis are given in Table 6.

Other factors that were associated with TTR were T stage (95% in T1, 87% in T2, 71% in T3, 42% in T4; p < 0.001), number of LNMs (83% in tumors with 0 LNM, 57% in tumors with 1-3 LNM, 32% in tumors with more than 3 LNM; p< 0.001), presence of intestinal obstruction (60% vs. 74%; p< 0.001), presence of tumor perforation (51% vs. 74%; p< 0.001), lymphovascular infiltration (45% vs. 78%; p< 0.003), perineural infiltration (49% vs. 77%; p< 0.001), and poor grade of differentiation (58 vs. 75%; p<0.001) (Table 6).

### Time to recurrence categorized by the presence of tumor nodes and lymph node metastasis

**Figure 6:**
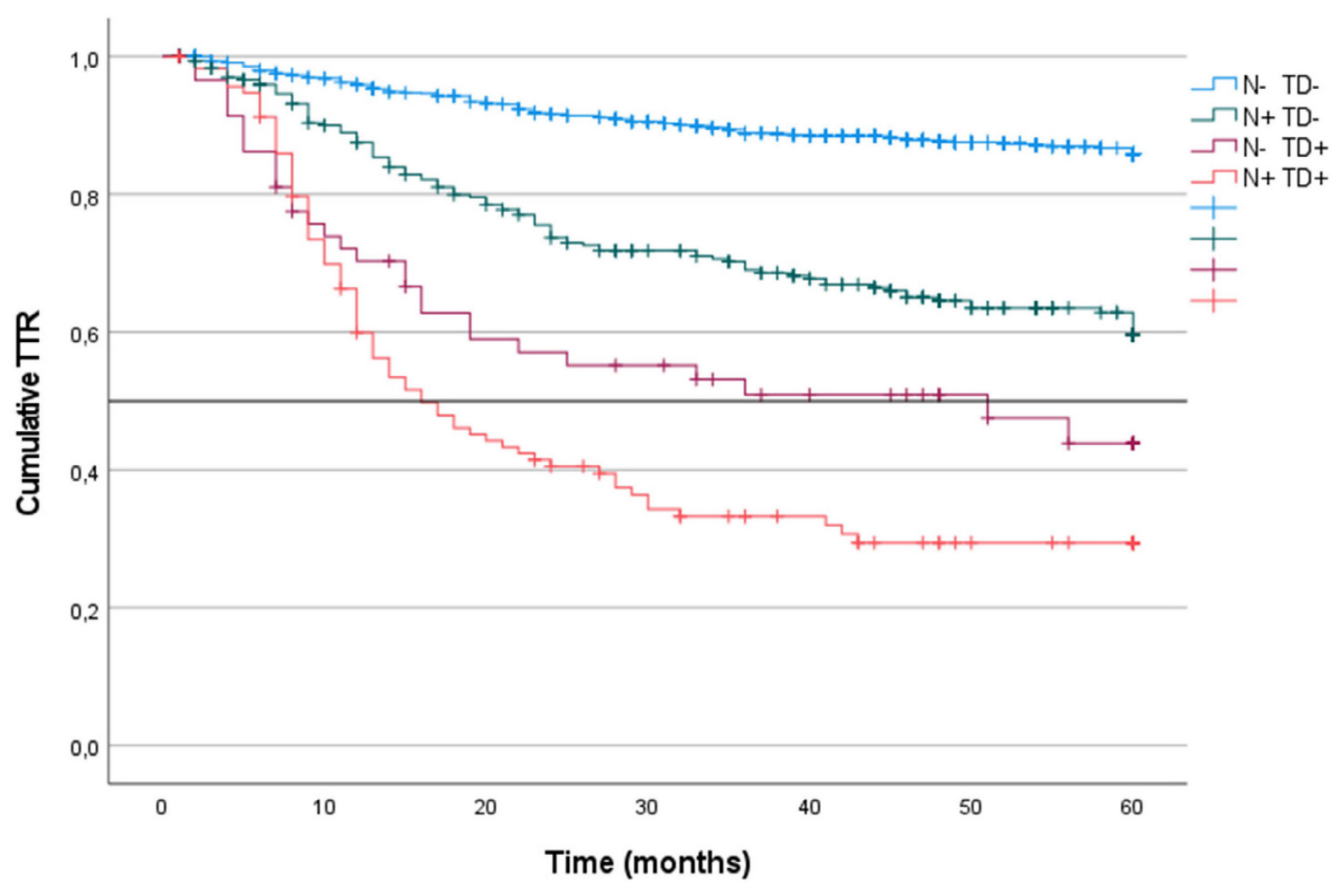
Kaplan-Meier estimates of TTR for the entire cohort according to the presence of Tumor Deposit and Lymph Node Metastasis. Horizontal bar denotes median Survival.

The TTR was analyzed at 60 months in the four groups of patients in the entire cohort according to the presence or absence of LNM and TD. Patients with TD^-^ or LNM^-^ tumors (85%) had a greater TTR than did patients with LNM^+^/TD^-^ tumors (59%) or TD^+^/LNM^-^ tumors (43%; mean: 51). The LNM^+^/TD^+^ patients had the lowest TTR (29%; mean: 16) (p < 0.001) (Figure 6).

We analyzed the TTR at 60 months of follow-up in the three subgroups of patients defined within category N1 according to the definition of the 8th edition of the AJCC TNM system. TTRs were significantly lower in N1c patients (44%; mean: 51) than in N1a patients (59%) and N1b patients (54%) (p = 0.033) (Table 7).

We analyzed the TTR of patients who were LNM^+^ classified according to the presence or absence of TD. In the N1 patients as a whole, the TTR was lower among the cases that presented LNM^+^/TD^+^ (39%; mean: 28) than in the LNM^+^/TD^-^ patients (63%) (p < 0.001) (Figure 6). In the N1a subgroup, the TTR was lower in the TD^+^ patients (45%; median 43) than in the TD^-^ patients (62%) (p = 0.020). Similarly, in N1b patients, the TTR was lower in TD^+^ patients (27%; median: 16) than in TD^-^ patients (65%) (p < 0.001) (Table 7).

**Figure 7:**
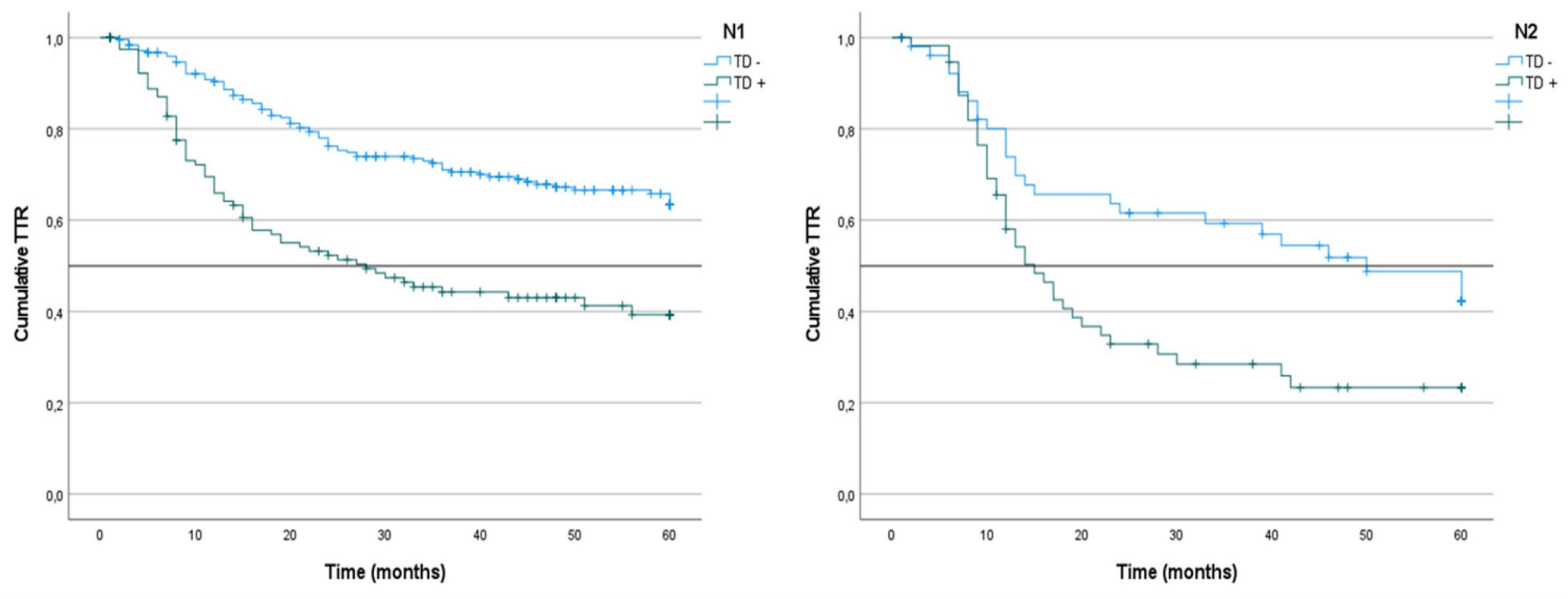
Kaplan-Meier estimates of TTR in N1 and N2 tumors according to presence of Tumor Deposit. Horizontal bars denote median Survival.

In N2 patients, the TTR was lower among the TD^+^LNM^+^/TD^+^ patients (23%; mean 15) than among the LNM^+^/TD^-^ patients (42%; mean: 50) (p = 0.008) (Figure 7). Among the N2a patients, the TTR was lower in patients with TD^+^ tumors, although the difference was not significant (36%; median: 12 vs. 42%; median: 50) (p = 0.276). Similarly, in N2b patients, the TTR was also lower among TD^+^ patients (20%; median: 16 vs. 41%; median: 41) (p = 0.053) (Table 7).

According to our multiple regression analysis, the presence of TDs was associated with a significant adverse effect on the TTR (HR=2.315; 95% CI: 1.743-3.073; p< 0.001) (Table 8). The other factors that showed an independent predictive value are shown in Table 8. The negative effect of LNM on the prognosis of TTR had a HR of 2.455 (95% CI: 1.719-3.507) in patients with 1-3 vs. 0 LNMs and 2.061 (95% CI: 1.588-2.676) in patients with > 3 vs. 1-3 LNMs.

In addition, we analyzed separately the effect of TDs on TTR in the group of TNM Stage III patients (Table 9). The multiple regression analysis, showed that in this group TDs had, also, a significant adverse effect on CRS (HR: 1.902; 95% CI: 1.411-2.950).

## Discussion

The results obtained in this study confirm that a negative prognosis is associated with the presence of TDs in patients with CRC. Patients with TD^+^ tumors had lower CRSs and TTRs at 60 months after diagnosis. According to our multiple regression analysis, the presence of TD was associated with an increased risk of death from CRC (HR: 1,820; 95% CI: 1,327-2,496) and an increased risk of recurrence (HR: 2.315; 95% CI: 1,743-3,073). Although the prognostic value of the presence of TDs was lower than that of LNM, the TD density had independent prognostic value and provided complementary information to that brought by LNM.

We found that patients with LNM^+^/TD^-^ and patients with LNM^-^/TD^++^ presented comparable CRS and TTR (CRS: 63% and 62%; TTR: 59% and 43%, respectively). Patients with tumors in which both factors were present had the lowest survival (36%; median: 41) (p < 0.001) and the lowest TTR (29%; median: 16) (p < 0.001). On the other hand, in each category of N stage, the CRS and TTR were lower in the patients who had TDs, which allowed us to identify two subgroups of patients differentiated by each of CRS and TTR.

The interest for the presence of TD in CRC is not new. This mater has been extensively investigated since 1997, when the AJCC adopted TD as a staging factor. TD has been gaining importance due to its prognostic value and subsequent implications in staging and treatment of CRC, which is shown by the high number of studies published during the last years. However, the pathobiological significance is still poorly understood, and its possible inclusion in the TNM classification system is still controversial. Our study has been performed following of definition of TD indicated by 8th edition of the AJCC TNM staging system. There are two publications designed according to those criteria 14,15. We coincide with those publications that the incidence of TD in non-metastatic CRC (9-12%) is lower than that found in studies developed following the previous criteria defined in the 7th edition of the TNM system (7%-35%, dependent on the studied population; average 22%) 2,5. In addition, we have studied a clinical series of patients and not a population-based cohort, which allows a closer follow-up and a more detailed collection of clinical data. This fact enabled to analyze concurrently survival and recurrence”.

LNM and TD were the histopathological variables that had the greatest independent prognostic weight in our study, which supports the inclusion of both variables in the definition of N-stage in the TNM system. The discussion ought to be on how to include TDs in that classification. The current system only considers TD when LNM does not coexist, which, in view of the results obtained, is insufficient. The TDs provide information independent of that provided by LNM and that is complementary. We verified that the determination of TDs allows the identification of risk subgroups within each N stage category that are not identified by the current system and characterized by having differentiated CRS and TTR. According to the 8th edition of the TNM system, a patient with a tumor containing 1-3 LNMs but without TD should be classified as the N1, as would another patient with the same number of LNMs but with TD^+^, which, as we have shown, is not appropriate. According to our data, the prognosis of patients in the latter group was closer to that of patients in the N2b subgroup. Additionally, a tumor with 4 or more LNMs but with a TD^+^ status was classified as N2b when the survival observed in our study was more typical of a stage IV tumor.

These data coincide with what has been published by several authors who, like us, have evaluated the prognostic value of TDs by classifying them in a categorical way (presence or absence) 6,15-18. Therefore, it has been proposed to modify the current classification system so that two subgroups are considered in each category of N stage (patients with or without TD). Pei 19 compared the predictive results provided by a new classification system carried out according to this principle and the 7th edition of the TNM system. The new classification showed greater prognostic power (AUC = 0.628, 95% CI=0.616-0.640) than did the TNM classification (AUC = 0.618; 95% CI=0.606-0.630) (p = 0.006). Furthermore, the creation of a new category called category N2c or N3 has been proposed; this category includes patients in which both factors coexist 13,20.

Other authors have reported that quantifying the number of TDs can provide more information than can determine the presence or absence 5,9,21,22. These authors proposed a new way to classify category N, in which the total sum of the number of deposits and the number of metastasized nodes was considered. Thus, a patient with 3 LNMs and a deposit, which would be classified, according to the current TNM system, as N1a, would progress, according to this proposal, to N2a. In three publications based on retrospective data 11,21,23, the new N category based on the “quantitative approach” provided better prediction of DFS and OS than the N category collected in the current TNM system. The results of two studies in which the prognostic value of TDs was analyzed on data from phase III clinical trials are also known 24,25. The patients in whom the N stage was modified from N1 to N2 according to the number of TDs and LNMs had significantly shorter DFS than the N1 patients, and the DFS was comparable to that of patients initially classified as N2. A retrospective study carried out with 500 stage III patients revealed that the number of TDs was correlated with DFS and OS 26. In this study, four groups of patients were defined according to the combination of TD and LNM, and a new category, N3, was incorporated, which included tumors with more than 10 TD^+^ LNMs. The three-year DFS was 86% in the N1, 74% in N2a, 58% in N2b, and 39% in N3 (p < 0.001).

The authors who have evaluated the results of this “quantitative” approach recognize that the differences between TDs and LNMs are evident in multiple ways, such as anatomical distribution, biological aggressiveness of the primary tumor, and predictive value. They do agree that the sum of the number of TDs and LNMs provides a better estimate of the evolutionary prognosis than the current classification 2.

Two other publications have compared the results of the two proposals for a new definition of category N 16,27. The evaluation of the number of TDs provided more information than that provided only by their presence/absence. In Liu's study 18, the OS of patients with 1 TD was slightly worse than that of patients with 1 LNM (p = 0.02), but no differences were found when 2 or more TDs were present. We were not able to include the number of TDs as a study variable, so we cannot support one option over another. We believe that the categorical classification of the presence/absence of TDs provides valuable predictive information that allows us to differentiate risk subgroups within each of the N categories that are not identified by the current TNM system. The presence of TD provides an added risk, so patients who meet these two criteria should move to a higher risk category. We consider Lino-Silva's proposal 20 to create a category N3, which includes patients who are currently classified as N2b but have TD^+^, and that patients now classified as N1b but with TD^+^ should now be considered N2b. More prospective studies are needed to determine how to integrate TDs into category N of the TNM system in patients who have both TDs and LNMs.

TDs are carcinoma foci separated from the primary tumor and located in the lymphatic drainage area. It is thought that these differences may be due to various causes, such as discontinuous local infiltration or lymphovascular or perineural spread. These lesions may represent lymph nodes or vascular or nerve structures completely filled with carcinoma. TDs are a histopathological marker that reflects the degree to which a tumor is aggressive, possibly through a greater capacity for migrating or infiltrating neighboring mesenchymal tissues. This means that patients with TD^+^ tumors have a lower survival rate and a higher incidence of recurrence 2.

## Conclusions

The results of our study showed that the presence of TDs was associated with a negative evolutionary course in patients with CRC, characterized by a lower CRS and TTR at 60 months after diagnosis. The predictive information derived from TD complements that provided by the presence of LNM and allows the identification of two subgroups of patients in each N stage category, CRS and TTR. The TD should be included in the definition of the TNM system in patients who simultaneously present with LNM.

## Figures and Tables

**Table 1:** Patient and tumor characteristics categorised by presence of Tumor Deposit

	NUMBER OF PATIENTS (n=1426)	TUMOR DEPOSIT NEGATIVE (n=1248)	TUMOR DEPOSIT POSITIVE (n=178)	P value
SEX				0.318
Women	542	471 (86.9%)	71 (13.1%)	
Men	884	777 (87.9%)	107 (12.1%)	
TUMOR SITE				0.885
Right Colon	471	413 (87.8%)	58 (12.3%)	
Left Colon	576	506 (87.8%)	70 (12.2%)	
Rectum	379	329 (86.8%)	50 (13.2%)	
AGE (years)				0.333
<50	105	94 (89.5%)	11 (10.5%)	
50-69	609	540 (88.6%)	69 (11.4%)	
>69	712	614 (86.2%)	98 (13.7%)	
T STAGE				<0.001
T1	161	160 (99.4)	1 (0.6)	
T2	246	239 (97.2)	7 (2.8)	
T3	845	729 (86.3)	116 (13.7)	
T4	174	120 (68.9%)	54 (31.1%)	
LYMPH NODE METASTASIS				<0.001
0	994	934 (93.9%)	60 (6.1%)	
1-3	319	259 (81.2%)	60 (18.8%)	
>3	113	55 (48.7%)	58 (51.3%)	
TUMOR GRADE				<0.001
Well-Moderately Differentiated	1317	1168 (88.7%)	149 (11.3%)	
Poorly Differentiated	109	80 (73.4%)	29 (26.6%)	
LYMPHOVASCULAR INFILTRATION				<0.001
No	1202	1112 (92.5%)	90 (7.5%)	
Yes	224	136 (60.7%)	88 (39.3%)	
PERINEURAL INFILTRATION				<0.001
No	1225	1124 (91.7%)	101 (8.3%)	
Yes	201	124 (61.7%)	77 (38.3%)	
HISTOLOGIC TYPE				0.098
Adenocarcinoma	1292	1136 (88%)	156 (12%)	
Mucinous	134	112 (83.6%)	22 (16.4%)	
INTESTINAL OBSTRUCTION				0.041
Absent	1304	1148 (88%)	156 (12%)	
Present	122	100 (82%)	22 (18%)	
TUMOR PERFORATION				<0.001
Absent	1360	1202 (88.4%)	158 (12.6%)	
Present	66	46 (69.7%)	20 (30.7%)	
POSTOPERATIVE ADJUVANT CHEMOTHERAPY				<0.001
No	796	743 (93.3%)	53 (6.7%)	
Yes	623	513 (82.3%)	110 (17.7%)	

χ2 test was used to calculate the P‑values.

**Table 2:** Tumor characteristics and Kaplan-Meier estimates of Cancer Related Survival at 60 months after diagnosis.

	PATIENTS (n)	CANCER RELATED SURVIVAL (60 months)	P VALUE	HAZARD RATIO	95% CI
AGE (years)			<0.001		
>70	712	71		1	
50-69	609	82		0.559	0.334-0.934
<50	105	81		0.528	0.407-0.686
SEX			0.496		
Male	884	76		1	
Female	542	79		0.917	0.712-1.179
TUMOR LOCALIZATION			0.073		
Right Colon	471	74		1	
Left Colon	576	79		0.747	0.546-1.020
Rectum	379	79		0.751	0.568-0.993
T STAGE			<0.001		
T1	161	96		1	
T2	246	89		17.349	7.130-43.691
T3	845	76		6.436	2.644-15.670
T4	174	48		2.629	0.987-7.006
LYMPH NODE METASTASIS			<0.001		
0	994	87		1	
1-3	319	63		5.987	4.339-8.261
>3	113	39		2.896	2.202-3.811
INTESTINAL OBSTRUCTION			<0.001		
Absent	1304	78		1	
Present	122	62		2.075	1.465-2.938
TUMOR PERFORATION			<0.001		
Absent	66	79		1	
Present	1360	46		3.853	2.632-5.639
TUMOR DEPOSIT			<0.001		
Absent	1248	82		1	
Present	178	42		4.497	4.366-5.835
LYMPHOVASCULAR INFILTRATION			<0.001		
Absent	1202	82		1	
Present	224	51		3.567	2.765-4.603
PERINEURAL INFILTRATION			<0.001		
Absent	1225	81		1	
Present	201	56		2.907	2.223-3.802
HISTOLOGIC TUMOR TYPE			0.015		
Classical Adenocarcinoma	1292	78		1	
Mucinous Carcinoma	134	71		1.558	1.086-2.233
GRADE OF DIFFERENTIATION			<0.001		
Well-Moderately Differentiated	1317	78		1	
Poorly Differentiated	109	60		2.410	1.702-3.413
POSTOPERATIVE ADJUVANT CHEMOTHERAPY			0.304		
No	796	78		1	
Yes	623	77		1.136	0.890-1.499

95% CI: 95% Confidence Interval

**Table 3:** Kaplan-Meier estimates of Overall Survival in N1 and N2 subgroups categorized by presence/absence of Tumor Deposits

	CRS ACCORDING TO TNM SYSTEM		CRS CATEGORIZED BY PRESENCE/ABSENCE OF TD				
			TD positive		TD Negative		
	CRS	median	CRS	median	CRS	median	P value
**N1**	63	-	51	-	68	-	<0.001
**N2**	39	51	24	33	56	-	<0.001
							
**N1a**	67	-	44	54	70	-	0.019
**N1b**	57	-	36	40	66	-	<0.001
**N1c**	63	-	63	-	-	-	-
							
**N2a**	51	-	34	18	58	-	0.012
**N2b**	34	46	23	33	53	-	0.031

**Table 4:** Predictive factors of Cancer Related Survival analyzed using Cox's proportional hazards model.

	P VALUE	HR	95% CI
AGE	<0.001		
>70		1	
50-69		0.434	0.256-0.736
<50		0.530	0.405-0.692
TUMOR LOCALIZATION	0.034		
Right Colon		1	
Left Colon		1.045	0.755-1.446
Rectum		0.722	0.541-0.962
T STAGE	<0.001		
T1		1	
T2		7.324	2.878-18.635
T3		3.739	1.519-9.201
T4		2.362	0.886-6.300
LYMPH NODE METASTASIS	<0.001		
0		1	
1-3		2.661	1.804-3.926
>3		1.961	1.466-2.622
TUMOR DEPOSIT	<0.001	1.820	1.327-2.496
LYMPHOVASCULAR INFILTRATION	0.001	1.628	1.208-2.195
INTESTINAL OBSTRUCTION	0.003	1.751	1.208-2.538
TUMOR PERFORATION	0.001	2.086	1.363-3.194

HR: Hazard Ratio, 95% CI: 95% Confidence Interval

**Table 5:** Prognostic factors of Cancer Related Survival in TNM Stage III patients analysed using Cox´s proportional hazards model

	P VALUE	HR	95% CI
AGE	<0.001		
>70		1	
50-69		0.506	0,282-0.910
<50		0.482	0.345-0.674
T STAGE	0.002		
T1		1	
T2		4.322	0.588-31.784
T3		2.156	0.298-15.577
T4		2.545	0.327-19.795
LYMPH NODE METASTASIS	0.003		
0		1	
1-3		2.628	1.474-4.687
>3		1,927	1.087-3.415
TUMOR DEPOSIT	<0.001	2.040	1.411-2.950
LYMPHOVASCULAR INFILTRATION	0.014	1.536	1.091-2.161

HR: Hazard Ratio, 95% CI: 95% Confidence Interval

**Table 6:** Tumor characteristics and Kaplan-Meier estimates of Time to Recurrence Survival at 60 months after diagnosis.

	PATIENTS (n)	TIME TO RECURRENCE (60 months)	P value	Hazard Ratio	95% CI
AGE (years)			0.107		
>70	712	71		1	
50-69	609	76		0.869	0.571-1.324
<50	105	75		0.782	0.624-0.984
SEX			0.142		
Male	884	71		1	
Female	542	76		0.842	0.668-1.060
TUMOR SITE			0.234		
Right Colon	471	73		1	
Left Colon	576	75		1.101	0.836-1.450
Rectum	379	71		0.879	0.676-1.143
T STAGE			<0.001		
T1	161	95		1	
T2	246	87		18.573	8.103-42.571
T3	845	71		7.050	3.131-15.876
T4	174	42		2.862	1.178-6.953
LYMPH NODE METASTASIS			<0.001		
0	994	83		1	
1-3	319	57		6.224	4.635-8.358
>3	113	32		2.397	2.397-3.931
INTESTINAL OBSTRUCTION			0.003		
Present	1304	74		1	
Absent	122	60		1.680	1.193-2.367
TUMOR PERFORATION			<0.001		
No	1360	74		1	
Yes	66	51		2.582	1.689-3.947
TUMOR DEPOSIT			<0.001		
Absent	1248	79		1	
Present	178	34		5.172	4.082-6.551
LYMPHOVASCULAR INFILTRATION			<0.001		
Absent	1202	78		1	
Present	224	45		3.532	2.794-4.464
PERINEURAL INFILTRATION			<0.001		
Absent	1225	77		1	
Present	201	49		3.058	2.393-3.907
HISTOLOGIC TUMOR TYPE			0.273		
Classical Adenocarcinoma	1292	74		1	
Mucinous Carcinoma	134	70		1.219	0.854-1.739
GRADE OF DIFFERENTIATION			<0.001		
Well-Moderately Differentiated	1317	75		1	
Poorly Differentiated	109	58		2.093	1.497-2.962
POSTOPERATIVE ADJUVANT CHEMOTHERAPY			<0.001		
Yes	623	67		1	
No	796	79		0.627	0.502-0.783

HR: Hazard Ratio. 95%CI: 95% Confidence Interval

**Table 7:** Kaplan-Meier estimates of TTR in N1 and N2 subgroups categorized by presence/absence of Tumor Deposits

	CRS ACCORDING TO TNM SYSTEM		CRS CATEGORIZED BY PRESENCE/ABSENCE OF TD				
			TD positive		TD negative		
	TTR	Median	TTR	Median	TTR	Median	P value
**N1**	55	-	39	28	63	-	<0.001
**N2**	31	22	23	15	42	50	0.008
							
**N1a**	59	-	45	43	62	-	0.020
**N1b**	54	-	27	16	65	-	<0.001
**N1c**	44	51	44	51	-	-	-
							
**N2a**	39	60	36	12	42	60	0.276
**N2b**	27	17	20	16	41	41	0.053

**Table 8:** Predictive factors of Time to Recurrence Survival analyzed using Cox's proportional hazards model.

	P value	HR	95% CI
TUMOR LOCALIZATION	0.002		
Right Colon		1	
Left Colon		1.333	1.005-1.767
Rectum		0.820	0.628-1.070
T STAGE	<0.001		
T1		1	
T2		8.942	3.825-20.903
T3		4.569	2.014-10.365
T4		2.530	1.040-6.151
LYMPH NODE METASTASIS	<0.001		
0		1	
1-3		2.455	1.719-3.507
>3		2.061	1.588-2.676
TUMOR DEPOSIT	<0.001	2.315	1.743-3.073
LYMPHOVASCULAR INFILTRATION	0.005	1.492	1.353-2.674

HR: Hazard Ratio, 95% CI: 95% Confidence Interval

**Table 9:** Prognostic factors of Time to Recurrence in TNM Stage III analysed using Cox´s proportional hazards model

	P VALUE	HR	95% CI
T STAGE	0.007		
T1-2		1	
T3		2.368	1.232-4.549
T4		1.512	0.831-2.752
LYMPH NODE METASTASIS	0.121		
0		1	
1-3		1.460	0.905-2.354
>3		1.142	0.711-1.837
TUMOR DEPOSIT	<0.001	1.902	1.353-2.674
LYMPHOVASCULAR INFILTRATION	0.019	1.438	1.062-1.947

HR: Hazard Ratio, 95% CI: 95% Confidence Interval
